# Efficacy of levosimendan for acute decompensated heart failure with preserved ejection fraction in the elderly: a single-center retrospective analysis

**DOI:** 10.1186/s12872-025-05224-3

**Published:** 2025-11-26

**Authors:** Xiaohong Xu, Fangchao Lv, Chenkai Xu, Xiuxiu Lai

**Affiliations:** 1 Department of Cardiology, Zhejiang Hospital, Hangzhou, Zhejiang China; 2 Department of Geriatric Medicine, Geriatric Medicine Center, Zhejiang Provincial People’s Hospital, Affiliated People’s Hospital, Hangzhou Medical College, 158 Shangtang Road, Xiacheng District, Hangzhou, Zhejiang China

**Keywords:** Levosimendan, Heart failure with preserved ejection fraction (HFpEF), Echocardiography, Global longitudinal strain (GLS), E/e' ratio

## Abstract

**Aims:**

Current guidelines recommend medical treatment for heart failure with preserved ejection fraction (HFpEF) but do not address inotropic drug use during acute HF episodes. This real-world study aimed to evaluate the effects of levosimendan in elderly patients presenting with acute HFpEF.

**Methods:**

We retrospectively identified patients aged ≥ 65 years hospitalized with acute HFpEF at our institution. Using propensity score matching (PSM), 160 patients were selected. Echocardiographic parameters and B-type natriuretic peptide (BNP) levels were assessed before and after levosimendan administration. Rehospitalization for heart failure (HF) and all-cause mortality were compared during follow-up.

**Results:**

Multivariable analysis revealed significantly greater improvements in the levosimendan group for septal early diastolic mitral inflow velocity to mitral annular tissue velocity ratio (E/e’) (ΔE/e’: -2.4 ± 2.4 vs. -1.9 ± 2.1; t = -2.09, 95% CI: -0.76 to -0.02; p = 0.038) and New York Heart Association (NYHA) class [median change: -1 (-1 to -0.25) vs. -1(-1 to 0); p = 0.048]. No significant between-group differences were observed in ΔBNP, Δabsolute global longitudinal strain (ΔaGLS), Δleft ventricular ejection fraction (ΔLVEF), or hospital length of stay. Subgroup analyses demonstrated greater improvements in ΔaGLS and Δseptal E/e’ among patients with baseline aGLS < 16% or septal E/e’ ≥15. After a median follow-up of 12.5 months, composite outcomes of rehospitalization for HF and all-cause mortality did not differ significantly between groups (levosimendan 21 vs. control 23; Log-rank *p* = 0.095).

**Conclusions:**

For older patients with acute HFpEF combined with more comorbidities, although levosimendan may transiently improve hemodynamics and functional capacity in the acute phase, it fails to demonstrate long-term prognostic benefits. The clinical benefit of this drug may be limited to select patients, and its clinical application may lack cost-effectiveness for broader application.

## Introduction

Heart failure with preserved ejection fraction (HFpEF) represents a major heart failure (HF) subtype, accounting for approximately 50% of all heart failure cases [1]. Characterized by high prevalence, mortality, and hospital readmission rates, HFpEF affects ~ 3 million individuals in the US, over 60% of whom are aged ≥ 65 years [2]. As the predominant HF phenotype in the elderly, its incidence and prevalence rise with population aging; notably, the risk of developing HFpEF increases by 1.91-fold per additional decade of age [3].

HFpEF occurrence is strongly associated with increasing prevalence of comorbidities such as obesity, diabetes, atrial fibrillation (AF), and hypertension [1–3]. Due to its complex pathophysiology and heterogeneous clinical presentation, the diagnosis, prognostic assessment, and treatment of HFpEF remain challenging.

Echocardiographic evaluation of HFpEF focuses on assessing cardiac morphology and diastolic function [4]. Key diastolic parameters include: left atrial volume index (LAVI); mitral annular tissue velocity (e’) measured by tissue Doppler imaging (TDI); early diastolic mitral inflow velocity(E)/e’ ratio; and tricuspid regurgitation velocity. An E/e’ ratio ≥ 15 demonstrates high specificity but relatively low sensitivity for diagnosing HFpEF [5]. Despite normal or preserved left ventricular ejection fraction (LVEF), many HFpEF patients exhibit subclinical systolic dysfunction [6]. Global longitudinal strain (GLS) is an emerging echocardiographic technique, valued for its stability and reproducibility, providing a sensitive tool for assessing myocardial contractility [7, 8]. Compared to LVEF, GLS demonstrates superior diagnostic and prognostic value in HFpEF patients [5, 9]. Previous studies report abnormal absolute GLS (aGLS; defined as GLS < 16%) prevalence ranging from 39% to 82% in stable HFpEF cohorts and 75% to 82% among patients with acute HF [7–9].

The 2022 American Heart Association (AHA)/American College of Cardiology (ACC)/Heart Failure Society of America (HFSA) Heart Failure Guidelines [1] and the 2023 European Society of Cardiology (ESC) guidelines [10] establish sodium-glucose cotransporter 2 (SGLT-2) inhibitors as foundational therapy for HFpEF, while emphasizing comprehensive management of key comorbidities (e.g., hypertension, diabetes, obesity). Diuretics are reserved exclusively for congestion relief, whereas inotropic agents are excluded from the standard HFpEF therapeutic regimen.

In clinical practice, we have observed that inotropic agents—particularly levosimendan—can improve clinical symptoms in acute-phase HFpEF patients with suboptimal response to diuretics. As a novel inotrope with multimodal mechanisms, levosimendan enhances calcium sensitivity of myocardial contractile proteins without increasing intracellular calcium concentration. This results in improved systolic function while avoiding elevated myocardial oxygen demand and calcium overload-associated ventricular arrhythmias [11, 12].

Previous research on levosimendan has predominantly focused on patients with heart failure with reduced ejection fraction (HFrEF) [12–14], with limited data available for HFpEF, particularly among elderly patients hospitalized for acute HF.

We hypothesized that levosimendan might benefit elderly HFpEF patients experiencing acute HF episodes. To evaluate its real-world efficacy, we retrospectively identified patients aged ≥ 65 years hospitalized with acute HFpEF at our institution. By comparing echocardiographic parameters (including GLS and E/e’), B-type natriuretic peptide (BNP) levels and New York Heart Association (NYHA) class before and after levosimendan administration, and assessing HF rehospitalization rates and all-cause mortality during follow-up, we aimed to determine its therapeutic effect.

## Methods

### Patient selection

We conducted a retrospective cohort study using electronic health records (EHRs) to identify patients diagnosed with HF between January 1, 2020 and December 31, 2023. HFpEF was confirmed per established diagnostic criteria [15]: (a)LVEF ≥ 50%, (b) ≥ 65 years old, (c)elevated BNP (BNP ≥ 35 pg/mL in AF patients or ≥ 105 pg/ml in sinus rhythm patients), (d)symptoms of HF (NYHA class II-IV). Exclusion criteria comprised༚prior LVEF < 40%, specific HF etiologies (congenital heart disease, hypertrophic cardiomyopathy, severe valvular disease, restrictive cardiomyopathy), and Inadequate echocardiographic image quality.

The study protocol was approved by the Clinical Investigation Committee of Zhejiang Hospital (No. 2024–078 K) with waiver of informed consent and conducted in accordance with the Declaration of Helsinki.

### Levosimendan intervention protocol

In hemodynamically unstable HFpEF patients with suboptimal response to standard therapy (volume optimization, ventricular rate control), levosimendan was administered as salvage therapy for persistent hypotension (SBP < 90 mmHg), oliguria (< 0.5 mL/kg/h), or unresolved symptoms. Dosing regimen: 0.10–0.20 µg/kg/min continuous infusion for 24 h under hemodynamic monitoring.

### Outcomes

Our primary endpoints were changes in echocardiographic parameters and BNP levels from baseline to discharge. The secondary endpoint was HF rehospitalization and all-cause mortality during post-discharge follow-up.

### Study measurements

#### Echocardiography

Comprehensive transthoracic echocardiography was performed at baseline and pre-discharge using a Philips IE33 system (X5-1 transducer, software version QLab 13.0). Standardized measurements were acquired in accordance with the American Society of Echocardiography (ASE) guidelines. M-mode/2D parameters: end-diastolic interventricular septal thickness (IVSd), Left ventricular end-diastolic dimension (LVEDD), pulmonary artery systolic pressure (PASP) and LVEF. Diastolic function indices: left atrial diameter (LAD); e’, E/e’ ratio and tricuspid regurgitation velocity.

GLS assessments [16] were measured in apical two-chamber, three-chamber and four-chamber views using the CMQ software in QLab. Region of interet (ROI) should cover the inner and outer membranes of left ventricle, and CMQ software can automatically trace each myocardial segment and display the speckle tracking echocardiography of each ventricular wall. If the tracking quality is poor and the image tracing area is outside the myocardial wall, the position and width of the ROI should be adjusted manually.

#### Definition of abnormal GLS

Given the meta-analysis-reported variability in normal GLS thresholds [absolute GLS (aGLS) range: 15.1–22.1%] [17] and the absence of consensus, we adopted the 2020 ESC HFpEF diagnostic algorithm threshold of aGLS < 16% [5] to define impaired myocardial deformation.

#### Statistical analysis

We performed propensity score matching (PSM) to balance baseline characteristics between groups. Covariates incorporated into the propensity score model included clinically relevant confounders: sex, age, drinking, smoking, body mass index and history of hypertension. Matching was conducted using a 1:1 nearest-neighbor algorithm with a caliper width of 0.2 standard deviations of the logit propensity score.

Categorical variables were described as numbers (frequencies). Continuous variables were all tested for a normal distribution, with normal distribution variables expressed as mean (𝑋) ± standard deviation (SD) and non-normal as median (interquartile range, IQR). χ2 test was used for comparison of categorical variables, T-test for normally distributed continuous variables and Mann-Whitney U test for non-normal continuous variables. Baseline variables with a p < 0.1 and echocardiographic parameters (aGLS, LVEF, septal E/e’, PASP) are included as covariates as a covariate in the regression model. The Kaplan Meier curves was taken to visualize cumulative rehospitalization rates for HF and composite outcome rates between two groups over four years. Log-rank test was used to compare long-term outcomes. Subgroup analysis was performed according to aGLS, septal E/e’ and AF. *P* < 0.05 was considered to be of statistically significance with a two-sided test.

All the statistical analyses were operated by R version 4.3.1 (R Foundation for Statistical Computing, Vienna, Austria) and IBM SPSS version 26.0 (SPSS Inc., Chicago, IL, USA).

## Results

### Study sample

**Fig. 1:**
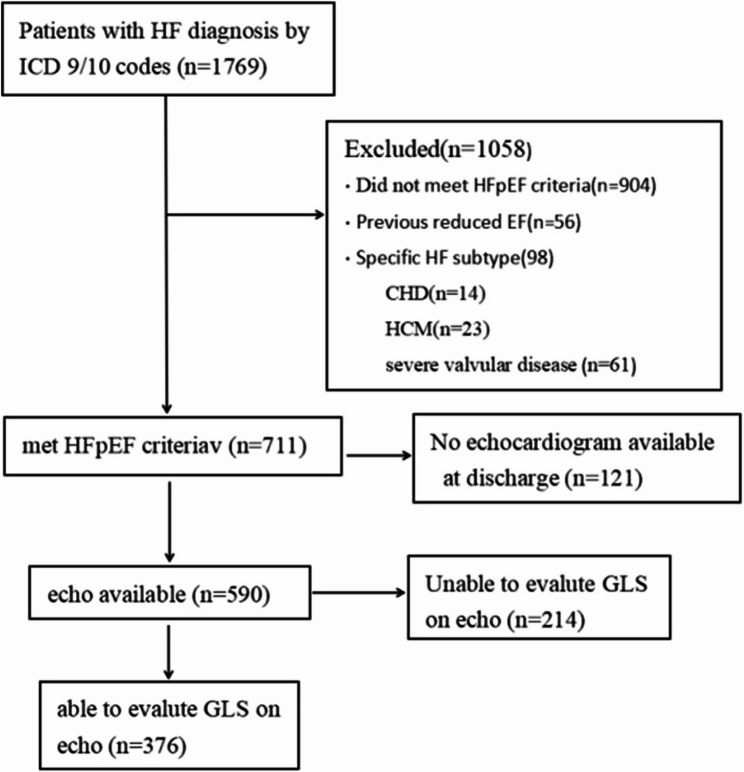
CONSORT Diagram. Abbreviation: ICD, international Classification of diseases; CHD, congenital heart disease; HCM, hypertrophic cardiomyopathy

From an initial screening of 1,769 acute decompensated HF inpatients (January 2020-December 2023), 904 patients were excluded for unmet diagnostic criteria and 489 for protocol-specified exclusion criteria (Fig. 1). The final cohort comprised 376 eligible patients, including 80 receiving levosimendan.

**Fig. 2:**
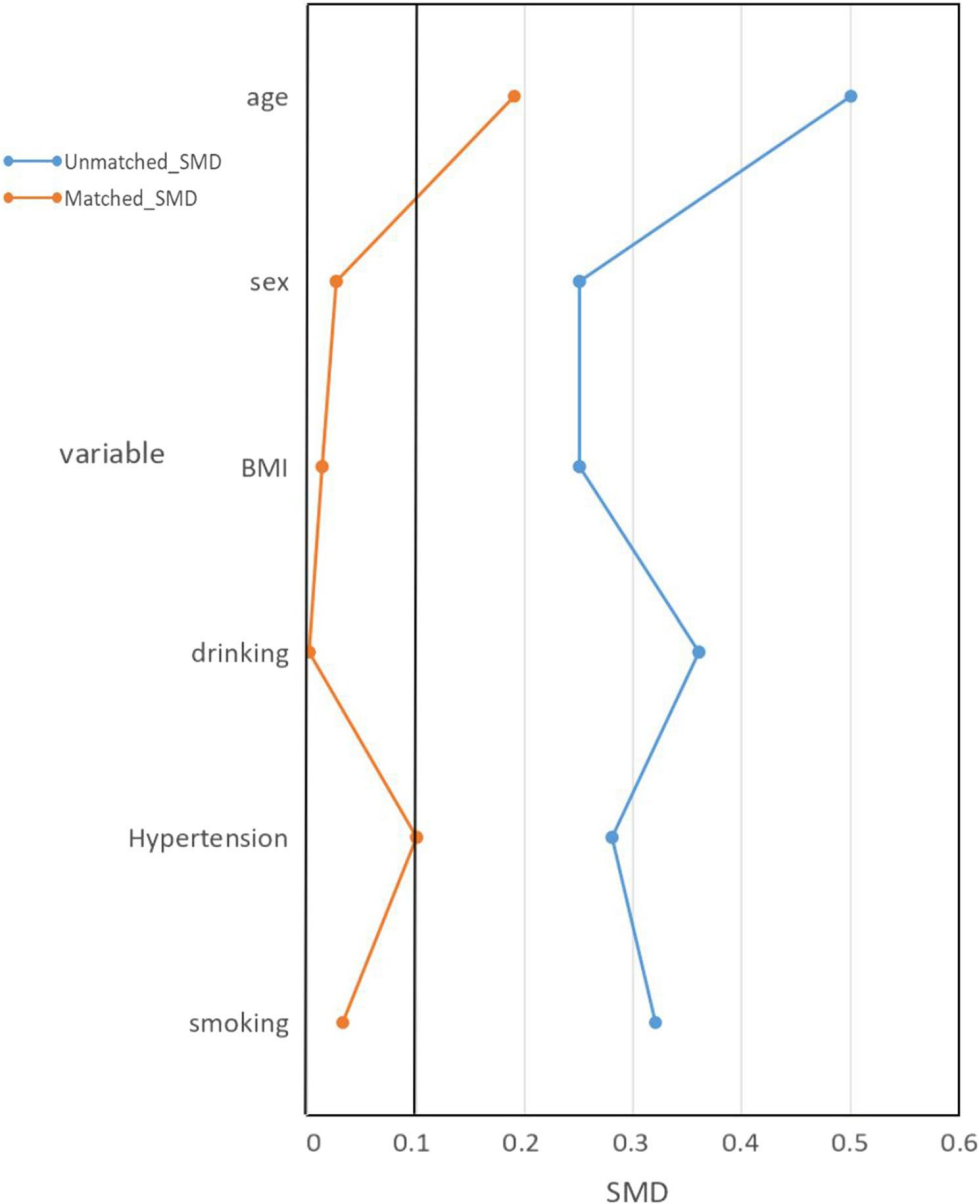
Propensity Score Matching. Abbreviation: BMI, body mass index; SMD, Standardized mean difference

To mitigate selection bias, 1:1 PSM was performed between levosimendan and non-levosimendan groups. Matching covariates included: age, sex, drinking, smoking, body mass index (BMI), hypertension history (defined as ≥ 140/90 mmHg or antihypertensive use).After matching, 80 propensity-matched controls were selected (Fig. 2). Baseline characteristics of the matched cohort are presented in Table 1.

**Table 1:** Baseline characteristics for matched study population

	Total Population *n* = 160	Levosimendan (+) *n* = 80	Levosimendan (-) *n* = 80	*p* value before PSM	*p* value after PSM	SMD after PSM
Age, years	86.0(79.0–90.0)	87.0(79.5–91.0)	84.0(79.0–88.0)	< 0.001	0.15	0.19
Male sex, *n* (%)	103(64.3%)	52(65.0%)	51(63.8%)	0.048	0.87	0.026
Smoking	29(18.2%)	14(17.5%)	15(18.8%)	0.011	0.84	0.032
Drinking	16(10.0%)	8(10.0%)	8(10.0%)	0.0045	1.0	< 0.001
BMI, kg/m2	24.3 ± 2.8	24.3 ± 2.7	24.4 ± 2.8	0.048	0.86	0.013
Blood Pressure, mmHg						
Systolic	138.6 ± 22.6	133.2 ± 22.9	144.1 ± 21.1	0.39	0.002	0.49
Diastolic	71.5(63.0–82.5.0.5)	71.0(63.5–81.0)	72.5(62.0–85.0)	0.33	0.78	0.034
HR, bpm	72.0(63.0–85.5.0.5)	76.8 ± 20.1	75.2 ± 17.0	0.65	0.59	0.086
Comorbidities						
* Acute infection*	84(52.5%)	42(52.5%)	42(52.5%)	0.29	1.0	< 0.001
* ACS*	26(16.3%)	8(10.0%)	18(22.5%)	0.037	0.032	0.34
CAD	111(69.4%)	54(67.5%)	57(71.3%)	0.30	0.61	0.081
OMI	13(8.1%)	9(11.3%)	4(5.0%)	0.13	0.15	0.23
Coronary Revascularization	24(15.0%)	6(7.5%)	18(22.5%)	0.12	0.008	0.43
Diabetes	64(40.0%)	33(41.3%)	31(38.8%)	0.15	0.75	0.051
Hypertension	135(84.4%)	66(82.5%)	69(86.3%)	0.027	0.51	0.10
Gout	10(6.3%)	4(5.0%)	6(7.5%)	0.71	0.51	0.10
AF	99(61.9%)	54(67.5%)	45(56.3%)	0.021	0.14	0.23
Stroke	27(16.9%)	14(17.5%)	13(16.3%)	0.69	0.83	0.033
CKD (stage IIb or higher)	82(51.3%)	43(53.8%)	39(48.8%)	0.019	0.53	0.10
Depression	20(12.5%)	14(17.5%)	6(7.5%)	0.003	0.056	0.31
Anemia	47(29.4%)	26(32.5%)	21(26.3%)	0.041	0.39	0.14
COPD	38(23.8%)	26(32.5%)	12(15.0%)	< 0.001	0.009	0.42
OSAS	1(0.6%)	0(0%)	1(1.3%)	0.64	0.32	0.16
Malignancy	9(5.6%)	3(3.8%)	6(7.5%)	0.34	0.30	0.16
NYHA Class				0.016	0.21	0.19
NYHA II	71(44.4%)	30(37.5%)	41(51.3%)	NA	NA	NA
NYHA III	73(45.6%)	41(51.3%)	32(40.0%)	NA	NA	NA
NYHA IV	16(10.0%)	9(11.3%)	7(8.8%)	NA	NA	NA
Prior LVEF (40–50%)	10(6.3%)	6(7.5%)	4(5.0%)	0.038	0.51	0.11
Laboratory Data						
Hemoglobin, mg/dL	117.0 ± 20.8	118.6 ± 22.2	115.3 ± 19.4	0.90	0.31	0.16
Sodium, mmol/L	139.5 ± 3.1	139.2 ± 3.0	139.8 ± 3.2	0.064	0.27	0.17
Potassium, mmol/L	4.2 ± 0.4	4.2 ± 0.4	4.1 ± 0.4	0.30	0.22	0.19
Creatinine, mg/dL	102.0(79.0–123.0.0.0)	106.5(84.8–125.0)	93.5(75.7–118.0)	0.77	0.07	0.13
BNP, pg/ml median (IQR)	547.0(315.7–820.2.7.2)	604.5(423.3–880.6.3.6)	496.4(260.9–771.6.9.6)	0.86	0.026	0.31
Fasting glucose	5.7(5.0–7.3.0.3)	5.7(5.0–7.7.0.7)	5.6(4.9–7.2)	0.095	0.33	0.071
Uric acid, µmol/l	393.4 ± 117.2	414.2 ± 118.9	372.6 ± 112.4	0.014	0.024	0.36
Echocardiographic Parameters						
LVEF (%)	59.0(54.7–65.4)	58.6(54.7–64.8)	59.7(54.3–66.8)	0.83	0.57	0.12
aGLS	16.4 ± 1.9	16.5 ± 1.8	16.5 ± 2.0	NA	0.91	0.018
LVEDD (mm)	47.8 ± 6.5	46.9 ± 6.3	48.7 ± 6.7	0.33	0.088	0.27
IVST (mm)	10.4(9.4–11.7)	10.5(9.4–12.2)	10.4(9.3–11.4)	0.069	0.33	0.16
Septal E/e’	13.4 ± 3.1	14.0 ± 3.1	12.8 ± 2.9	0.001	0.012	0.40
PASP	59.6 + 14.3	61.8 ± 13.0	57.5 ± 15.2	0.026	0.057	0.29
Drug Therapy						
Beta-Blocker	108(67.5%)	52(65.0%)	56(70.0%)	0.10	0.50	0.11
ACEI/ARB/ARNI	116(72.5%)	56(70.0%)	60(75.0%)	0.076	0.48	0.11
SGLT2-i	84(52.5%)	44(55.0%)	40(50.0%)	0.001	0.53	0.10
MRA	122(76.3%)	62(77.5%)	60(75%)	0.17	0.71	0.059
Loop Diuretic	157(98.1%)	80(100.0%)	77(96.3%)	0.002	0.08	0.28
Antiplatelet drugs	61(38.1%)	22(27.5%)	39(48.8%)	0.001	0.006	0.45
OACs	74(46.3%)	42(52.5%)	32(40.0%)	0.018	0.11	0.25
Statin	128(80.0%)	62(77.5%)	66(82.5%)	0.49	0.43	0.13

*Abbreviations*: *SMD* Standardized Mean Difference, *BMI* Body mass index, *HR* Heart rate, *ACS* Acute coronary syndrome, *CAD* Coronary artery disease, *OMI* Old Myocardial Infarction, *AF* Atrial Fibrillation/Flutter, *CKD* Chronic kidney disease, *COPD* Chronic obstructive pulmonary disease, *OSAS* Obstructive sleep apnea syndrome, *NYHA* New York Heart Association, *LVEF* Left ventricular ejection fraction, *BNP* B-type natriuretic peptide, *aGLS* Absolute global longitudinal strain, *LVEDD* Left ventricular end diastolic diameter, *IVST* Interventricular septal thickness, *E/e’* Early diastolic mitral inflow velocity/mitral annular tissue velocity by tissue Doppler imaging, *PASP* Pulmonary artery systolic pressure, *ACEI/ARB/ARNI* Angiotensin converting enzyme inhibitor/angiotensin receptor blocker/angiotensin receptor neprilysin inhibitor, *SGLT-2i* Sodium glucose cotransporter-2 inhibition, *MRA* Mineralocorticoid receptor antagonist, *OACs* Oral anticoagulants

### Baseline characteristics

The propensity-matched cohort (*N* = 160) comprised predominantly elderly patients (median age 86 years, IQR 79–90) with high multimorbidity burden (91.3% having ≥ 2 chronic conditions). Baseline characteristics demonstrated balance except for ACS, prior coronary revascularization, systolic blood pressure (SBP) and chronic obstructive pulmonary disease (COPD).

Laboratory parameters (Table 1) revealed comparable hemoglobin, electrolytes, creatinine, and fasting glucose levels; however, the levosimendan group exhibited elevated BNP [median 604.5(IQR 423.3–880.6.3.6)vs 496.4 (IQR 260.9–771.6.9.6)pg/mL, *p* = 0.026] and serum uric acid (414.2 ± 118.9 vs. 372.6 ± 112.4 µmol/L, *p* = 0.024).

Echocardiographic assessment showed overall median LVEF of 59.0% (IQR 54.7–65.4), impaired myocardial deformation (aGLS < 16%) in 38.8% of patients, and mean septal E/e’ of 13.4 ± 3.1 (70.6% with E/e’ ≥15). Despite comparable LVEF, aGLS and LVEDD, septal E/e’ was higher in the levosimendan group (14.0 ± 3.1 vs. 12.8 ± 2.9, *p* = 0.012).

All patients received guideline-directed medical therapy (GDMT). Concomitant medications showed no intergroup differences in Angiotensin-Converting Enzyme Inhibitors (ACEI)/Angiotensin Receptor Blockers (ARB)/angiotensin receptor neprilysin inhibitor (ARNI), SGLT2 inhibitors, mineralocorticoid receptor antagonist (MRA), β-blockers, loop diuretics, anticoagulants, or statins, though antiplatelet use was lower in levosimendan group (27.5% vs. 48.8%, *p* = 0.006), consistent with their reduced revascularization history.

### Outcomes

#### In-hospital outcomes

Univariate analysis demonstrated comparable changes from baseline to discharge in LVEF (levosimendan: 0.2 ± 2.3% vs control: 0.4 ± 1.8%; t=−2.5, p = 0.55) and septal E/e’ (levosimendan: −2.4 ± 2.4 vs. control: −1.9 ± 2.1; t = 1.45, *p* = 0.15). However, the levosimendan group exhibited significantly greater improvements in BNP reduction [median − 319.0 (IQR − 559.5 to −121.5) vs. −214.3 (IQR − 464.9 to 0) pg/mL; Z=−2.44, *p* = 0.015], aGLS increase (0.8 ± 1.0% vs. 0.4 ± 0.9%; t=−2.5, *p* = 0.013), and NYHA class improvement [median − 1 (IQR − 1 to −0.25) vs. −1 (IQR − 1 to 0); Z=−2.16, 0.031] (Table 2).

**Table 2:** Univariate analysis

	Levosimendan (+)				Levosimendan (-)				inter- group *p* value	t/Z value
	Baseline	Before discharge	Change	*p* value	Baseline	Before discharge	Change	*p* value		
aGLS (%)	16.5 ± 1.8	17.3 ± 1.5	0.8 ± 1.0	< 0.001	16.5 ± 2.0	16.9 ± 1.8	0.4 ± 0.9	< 0.001	0.013	−2.5
LVEF (%)	58.6 (54.7 to 64.8)	58.4 (56.1 to 64.8)	0.2 ± 2.3	0.56*	59.7 (54.3 to 66.8)	60.9 (55.4 to 66.3)	0.4 ± 1.8	0.051*	0.55	0.60
septal E/e’	14.0 ± 3.1	11.6 ± 1.4	−2.4 ± 2.4	< 0.001	12.8 ± 2.9	10.9 ± 1.5	−1.9 ± 2.1	< 0.001	0.15	1.45
PASP	61.8 ± 13.0	32.6 ± 6.7	−26.2 ± 11.7	< 0.001	57.5 ± 15.2	37.4 ± 12.5	−20.0 ± 15.4	< 0.001	0.005	2.82
BNP, pg/ml	604.5 (423.3 to 880.6)	302.9 (182.2 to 426.8)	−319.0 (−559.5 to-121.5)	< 0.001*	496.4 (260.9 to771.6)	242.8 (158.0 to 381.6)	−214.3 (−464.9 to 0)	< 0.001*	0.015	−2.44#
NYHA class	3 (2 to 3)	2(2 to 2)	−1(−1 to −0.25)	< 0.001*	2 (2 to 3)	2(1 to 2)	−1 (−1 to 0)	< 0.001*	0.031	−2.16#
Length of stay (days)	10.4 ± 4.7				9.3 ± 5.7				0.19	−1.32

*Abbreviations*: *aGLS* Absolute global longitudinal strain, *LVEF* Left ventricular ejection fraction, *E/e’* Early diastolic mitral inflow velocity/mitral annular tissue velocity by tissue Doppler imaging, *PASP* Pulmonary artery systolic pressure, *BNP* B-type natriuretic peptide, *NYHA* New York Heart Association. *Wilcoxon signed-rank test; #Z value for Mann-Whitney U test

Multivariable adjustment attenuated these differences, showing no significant between-group effects on LVEF, BNP, or aGLS changes (Table 3). A marginal benefit was observed for septal E/e’ reduction in the levosimendan group (−2.4 ± 2.4 vs −1.9 ± 2.1, t=−2.09;95% CI −0.76 to −0.02;*p* = 0.038) and NYHA improvement [−1(−1 to −0.25) vs. −1 (−1 to 0), t = 1.99; 95% CI 0.002 to 0.50; *p* = 0.048], though evidence strength remained limited.

**Table 3:** Multivariable analysis (Adjusted for SBP, ACS, coronary revascularization, depression, COPD, creatinine, BNP, uric acid and septal E/e’, aGLS, LVEF, PASP)

	Levosimendan (+)	Levosimendan (-)	t	Adjusted difference between groups (95% CI)	*p*
ΔaGLS	0.8 ± 1.0	0.4 ± 0.9	1.52	−0.55 to 0.051	0.10
ΔLVEF (%)	0.2 ± 2.3	0.4 ± 1.8	1.14	−0.30 to 1.12	0.26
Δseptal E/e’	−2.4 ± 2.4	−1.9 ± 2.1	−2.09	−0.77 to −0.02	0.039
ΔPASP	−26.2 ± 11.7	−20.0 ± 15.4	0.64	−2.28 to 4.43	0.53
ΔBNP	−319.0 (−559.5 to −121.5)	−214.3 (−464.9 to 0)	−1.00	−100.53 to 33.21	0.32
ΔNYHA class	−1(−1 to −0.25)	−1 (−1 to 0)	1.99	0.002 to 0.50	0.048
Length of stay (days)	10.4 ± 4.7	9.3 ± 5.7	−0.39	−2.00 to 1.34	0.70

*Abbreviations*: *SBP* Systolic Blood Pressure, *ACS* Acute coronary syndrome, *COPD* Chronic obstructive pulmonary disease, *BNP* B-type natriuretic peptide, *E/e’* Early diastolic mitral inflow velocity/mitral annular tissue velocity by tissue Doppler imaging, *aGLS*, Absolute global longitudinal strain, *LVEF* Left ventricular ejection fraction, *PASP* Pulmonary artery systolic pressure, *NYHA* New York Heart Association

#### Long-term outcomes

**Fig. 3:**
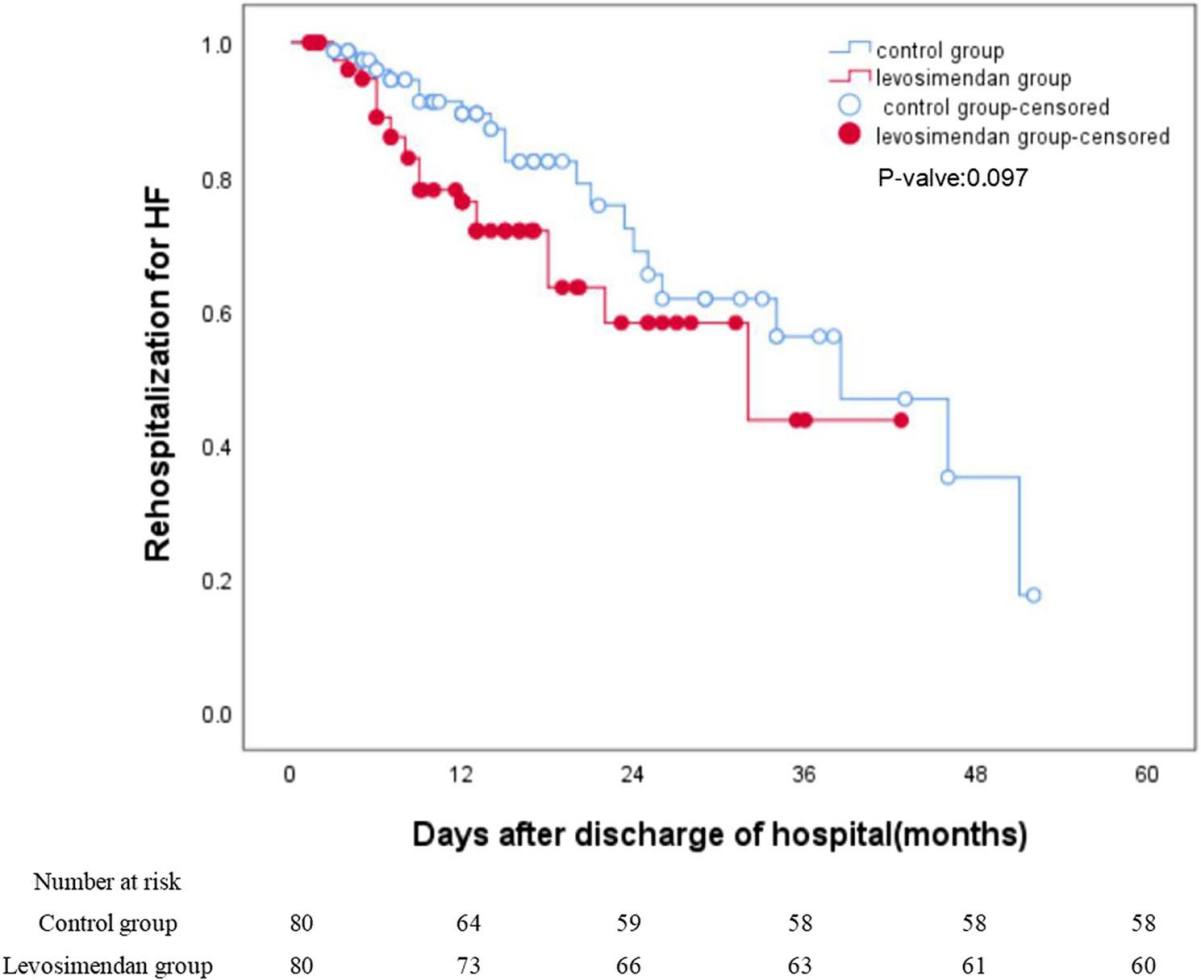
Unadjusted Kaplan-Meier Curves: Rehospitalization for HF. Abbreviation: HF, heart failure

**Fig. 4:**
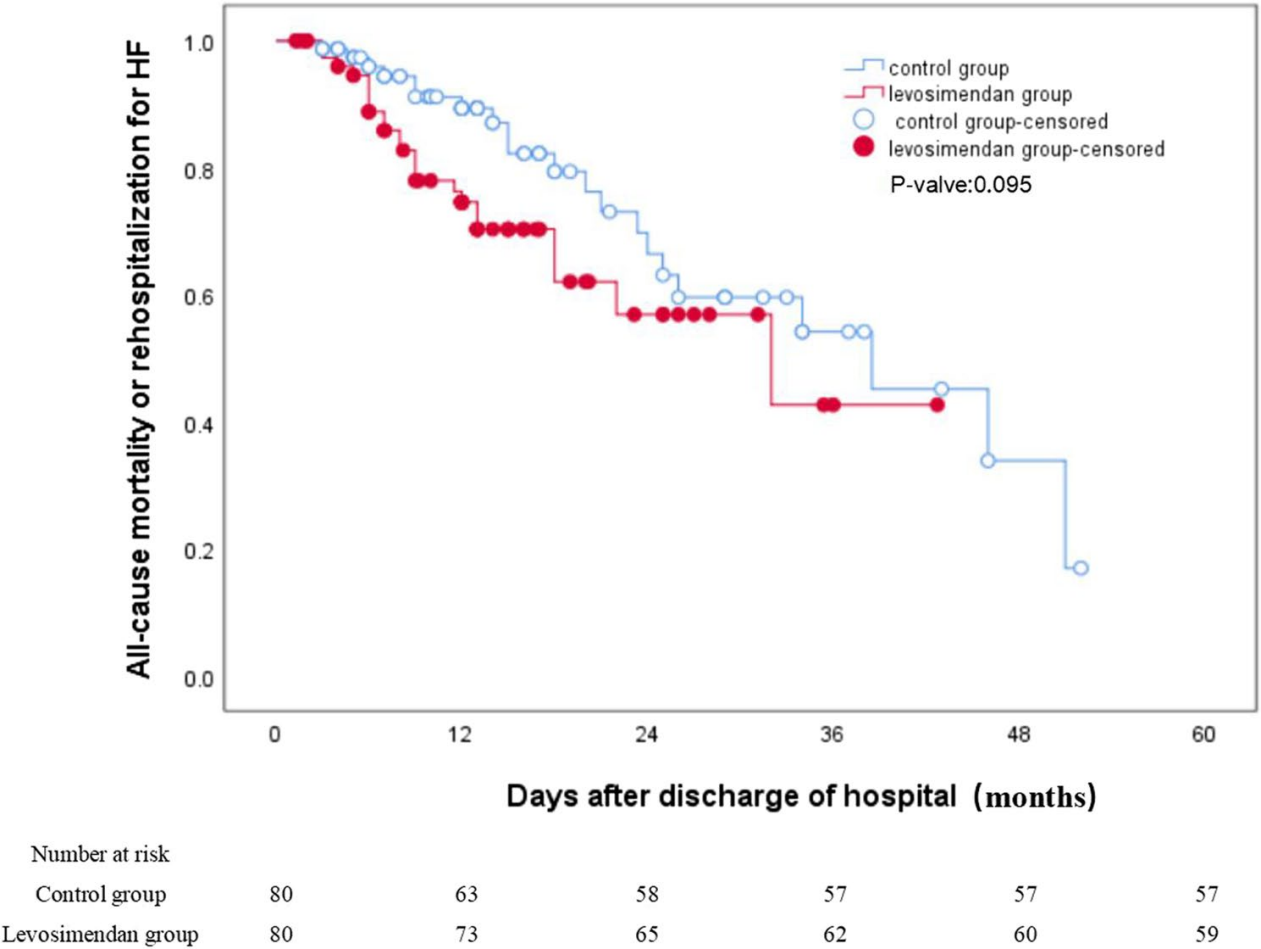
Unadjusted Kaplan-Meier Curves: All-cause mortality or rehospitalization for HF. Abbreviation: HF, heart failure

During a median follow-up of 12.5 months, HF rehospitalization occurred in 20 levosimendan-treated patients versus 22 controls (log-rank *p* = 0.097). The composite endpoint of HF rehospitalization or all-cause mortality also showed no significant difference (21 vs. 23 events; log-rank *p* = 0.095). Unadjusted Kaplan-Meier curves for both endpoints are presented in Fig. 3 (HF rehospitalization) and Fig. 4 (composite endpoint).

#### Subgroup analyses

Prespecified subgroup analyses stratified by aGLS < 16%, septal E/e’≥15 and AF revealed differential treatment responses (Table 4). Key findings demonstrated that patients with impaired myocardial deformation (aGLS < 16%) exhibited significantly greater improvements in septal E/e’ reduction (−3.98 ± 2.45 vs −1.56 ± 1.92; t=−4.87, p < 0.001), aGLS increase (1.52 ± 0.97% vs 0.42 ± 0.70%; t = 5.34, p < 0.001), and LVEF improvement (1.85 ± 2.00% vs −0.70 ± 1.87%; t = 5.69, p < 0.001). Furthermore, levosimendan provided pronounced E/e’ reduction in E/e’ ≥15 subgroup (−4.75 ± 1.93 vs −1.00 ± 1.32; t=−10.33, *p* < 0.001) and AF subgroup (−2.85 ± 2.50 vs −1.49 ± 1.92; t=−2.45, *p* = 0.016).

**Table 4:** Subgroup analysis among Levosimendan group

Outcomes	Subgroup		*n*	Change from baseline	t/z value	*p* for interaction
aGLS	aGLS < 16	yes	28	1.52 ± 0.97	5.34	< 0.001
		no	52	0.42 ± 0.70		
	E/e’≥15	yes	50	1.05 ± 0.98	2.211**	0.027
		no	30	0.4(0 to 1.22)*		
	AF	yes	54	0.84 ± 0.90	0.69	0.49
		no	26	0.73 ± 1.09		
LVEF	aGLS < 16	yes	28	1.85 ± 2.00	5.69	< 0.001
		no	52	−0.70 ± 1.87		
	E/e’≥15	yes	50	0.78 ± 2.12	1.84	0.070
		no	30	−0.16 ± 2.30		
	AF	yes	54	0.31 ± 2.22	0.68	0.50
		no	26	−0.06 ± 2.39		
Septal E/e’	aGLS < 16	yes	28	−3.98 ± 2.45	−4.87	< 0.001
		no	52	−1.56 ± 1.92		
	E/e’≥15	yes	50	−4.75 ± 1.93	−10.33	< 0.001
		no	30	−1.00 ± 1.32		
	AF	yes	54	−2.85 ± 2.50	−2.45	0.016
		no	26	−1.49 ± 1.92		
BNP	aGLS < 16	yes	28	−486.50 (−977.00 to −289.92) *	−0.48**	0.63
		no	52	−273.00 (−400.64 to −71.50) *		
	E/e’≥15	yes	50	−592.50 (−992.25 to −320.25) *	−0.30**	0.77
		no	30	*−218.0 (−341.00 to −70.00) *		
	AF	yes	54	−323.77 (−717.75 to −183.00) *	−1.96**	0.050
		no	26	−216.31 (−429.04 to −29.17) *		
Length of stay (days)	aGLS < 16	yes	28	10.33 ± 4.72	−1.53	0.13
		no	52	11.99 ± 4.56		
	E/e’≥15	yes	50	11.50 ± 4.92	0.22	0.83
		no	30	11.26 ± 4.25		
	AF	yes	54	10.49 ± 4.74	0.23	0.82
		no	26	10.23 ± 4.56		

*Abbreviations*: *aGLS* Absolute global longitudinal strain, *LVEF* Left ventricular ejection fraction, *E/e’* Early diastolic mitral inflow velocity/mitral annular tissue velocity by tissue Doppler imaging, *AF* Atrial fibrillation, *BNP* B-type natriuretic peptide; *: interquartile range; **: Z value for Mann Whitney U test

## Discussion

This retrospective study presents real-world data on levosimendan use in patients aged ≥ 65 years with acute HFpEF. Elderly patients differ markedly from younger cohorts in terms of physiological reserve and comorbidity profiles, often presenting with multiple chronic conditions and a heightened predisposition to myocardial fibrosis, vascular stiffness, and renal impairment—all contributing to left ventricular diastolic dysfunction [1]. Older adults with HFpEF frequently exhibit exertional dyspnea and reduced exercise tolerance, clinical features that may be mistaken for age-related frailty [18, 19]. Due to this nonspecific symptomatology, HFpEF in the elderly is often underdiagnosed. In our cohort, over 90% of patients had two or more chronic diseases, with hypertension, CAD, and AF being the most prevalent.

Recent advances have deepened our understanding of HFpEF’s heterogeneous etiology and complex pathophysiology [18, 19]. The syndrome is characterized by interactions among multiple risk factors and cardiac abnormalities, including left ventricular diastolic dysfunction, impaired long-axis systolic function, and atrial myopathy. These alterations collectively lead to elevated left ventricular end-diastolic pressure (LVEDP) and manifest as clinical HFpEF [6, 18, 19].

### Diagnosis and prognosis assessment

#### aGLS

Echocardiography remains the primary modality for evaluating cardiac structure and function [4]. Although current guidelines continue to classify heart failure (HF) phenotypes based on LVEF, with HFpEF defined as LVEF ≥ 50% [1], this parameter has notable limitations. LVEF measurement is subject to variability, with intra-individual deviations of up to 10%, and its cutoff values do not reliably distinguish between HF phenotypes.

Many HFpEF patients exhibit impaired systolic function despite preserved LVEF and apparent diastolic dysfunction. This subclinical impairment can be detected using aGLS [20], a sensitive marker of myocardial contractility [6–9], as it captures the subendocardial longitudinal fibers which are particularly vulnerable to ischemia, fibrosis, and elevated filling pressures—hallmarks of the HFpEF syndrome. This makes it superior to LVEF in detecting subtle systolic dysfunction that is otherwise obscured by preserved LVEF. Besides, aGLS is derived from speckle-tracking echocardiography and analyzed using automated software (e.g., CMQ in QLab, as used in this study), which improves reproducibility and reduces operator-dependent variability. However, its clinical adoption remains limited due to the time-consuming analysis process.

In healthy individuals, aGLS typically ranges between 17.1% and 21.5%, whereas HFpEF patients show reduced values, often between 12% and 18.9% [17]. The prevalence of impaired aGLS (< 16%) varies from 39% to 82% in chronic HFpEF cohorts and reaches 75–82% in acute HFpEF settings [7–9]. This impairment is strongly associated with adverse cardiovascular outcomes [21, 22]. For example, Donal et al. reported that acute HFpEF patients with aGLS < 16% had a significantly elevated risk of HF rehospitalization (HR = 1.94, *p* = 0.0047) [21]. Accordingly, the 2020 ESC guidelines endorse aGLS < 16% as a diagnostic criterion for HFpEF [5].

Furthermore, aGLS possesses prognostic utility for predicting HF progression in HFpEF [5, 22, 23]. Alison et al. [24] observed that patients with aGLS < 15.8% were more likely to experience LVEF decline (19% vs. 10%; HR 2.2, *p* = 0.018). Each 1% reduction in aGLS was associated with a 5% increased risk of all-cause mortality, a 10% higher risk of composite endpoint events, and a 13% greater likelihood of LVEF deterioration.

The findings align with a study by Buggey et al. [22] of 463 acute HFpEF patients, which reported a median aGLS of 12.8% (IQR: 10.8–15.8%). The lower aGLS in their cohort compared to ours (16.4 ± 1.9%) may reflect ethnic differences [25] and a higher comorbidity burden, including hypertension, chronic kidney disease, diabetes, and prior coronary revascularization. Our comparatively higher aGLS suggests a study population with less advanced myocardial impairment, underscoring the well-established heterogeneity of HFpEF and emphasizing the need to identify sub-phenotypes most likely to benefit from targeted therapies such as levosimendan, which enhances both contractility and lusitropy.

#### E/e’

In the evaluation of patients with HFpEF, assessing left ventricular diastolic function is crucial [4]. Commonly used echocardiographic parameters include LAVI, e′, the ratio of E/e′, and tricuspid regurgitation velocity. Among these, E/e′ has been shown to offer the highest diagnostic utility with relatively high specificity for detecting elevated left ventricular filling pressure. A systematic review indicated that E/e′ correlates modestly but significantly with invasively measured LV filling pressures [5, 26]. As a result, it is widely recommended as a noninvasive surrogate for estimating LV filling pressure due to its favorable accuracy and clinical feasibility [27, 28].

Notably, the 2020 Heart Failure Association of the ESC guidelines endorse an E/e′ ratio ≥ 15 as a key diagnostic criterion for HFpEF, prioritizing high specificity to rule in the condition despite acknowledging its limited sensitivity [5]. In our cohort, 70.6% of patients exhibited E/e′ ≥15, indicating that a substantial majority of elderly acute HFpEF patients had elevated LVEDP. This high prevalence underscores the severity of diastolic impairment in this demographic and reinforces the relevance of E/e′ as a critical marker in real-world clinical settings.

However, the interpretation of E/e′ in older adults requires caution. Age-related changes in myocardial relaxation and loading conditions can influence the ratio, and its accuracy may be reduced in certain subgroups, such as those with severe mitral annular calcification or AF. Nevertheless, its integration into a comprehensive diagnostic algorithm remains indispensable. The high proportion of abnormal E/e′ in our study not only validates its utility in phenotyping elderly HFpEF patients but also highlights its potential role in enriching future trial cohorts for interventions targeting diastolic dysfunction, such as levosimendan, which modulates lusitropy and may ameliorate high filling pressures.

### Levosimendan treatment

#### Mechanism of action of Levosimendan

Levosimendan is a multifaceted inotropic agent with a unique pharmacological profile that holds significant potential for the treatment of acute HF. Its mechanism of action is primarily dual: it enhances cardiac contractility without increasing intracellular calcium concentration, promotes systemic vasodilation [11, 12, 29–31].

Specifically, levosimendan selectively binds to cardiac troponin C, increasing the calcium sensitivity of contractile proteins. This calcium-sensitizing effect improves myocardial contractility while avoiding intracellular calcium overload and the associated risk of arrhythmias [11, 12]. Simultaneously, by opening ATP-sensitive potassium channels in vascular smooth muscle and mitochondria, levosimendan induces vasodilation, which reduces cardiac preload and afterload, improves coronary perfusion, and attenuates ischemia-reperfusion injury [29, 30]. Additionally, emerging evidence suggests that levosimendan may confer anti-inflammatory and anti-apoptotic benefits in patients with severe HF [31].

While calcium sensitization and vasodilation have traditionally been regarded as the cornerstone mechanisms of levosimendan [11, 12], recent studies propose a reinterpretation of its inotropic actions. Investigations in isolated human failing myocardium indicate that the positive inotropic effect of levosimendan—and its active metabolite, OR-1896—may be primarily mediated through phosphodiesterase III (PDE-III) inhibition, with no significant calcium-sensitizing effect observed in human tissue under experimental conditions [32, 33]. In contrast, studies in rat models have demonstrated calcium sensitization, highlighting a notable species-dependent discrepancy [33]. Consequently, some authors suggest that levosimendan should be reclassified primarily as a PDE-III inhibitor rather than a calcium sensitizer in the context of human HF [33].

#### Levosimendan for HFpEF

Research on levosimendan has primarily focused on patients with HFrEF [12–14], with limited studies conducted in those with HFpEF. The pathogenesis of HFpEF is multifactorial and complex [34], involving impairments in cellular calcium handling such as: (1) dysregulation of sarcolemmal calcium efflux pathways, including dysfunctional calcium pumps and sodium-calcium exchangers; (2) impaired sarcoplasmic reticulum calcium reuptake, largely due to reduced sarcoplasmic reticulum calcium ATPase (SERCA2a) activity; and (3) altered phosphorylation of key regulatory proteins such as phospholamban. Notably, SERCA2a expression declines with age, contributing to both systolic and diastolic dysfunction.

Given these mechanisms, levosimendan—a calcium sensitizer and vasodilator—represents a theoretically promising treatment for HFpEF. In the recent multicenter, double-blind, hemodynamic evaluation HELP trial involving HFpEF patients [35], weekly levosimendan infusion did not significantly reduce the primary endpoint of exercise pulmonary capillary wedge pressure (PCWP). However, it consistently lowered PCWP across all exercise levels and reduced central venous pressure at 6 weeks, while also improving 6-minute walking distance. Further analysis revealed a significant reduction in stressed blood volume (SBV), suggesting that even low-dose levosimendan (0.10 mg/kg/min) exerts venodilatory effects [35]. This aligns with Kass et al. [12], who proposed that levosimendan enhances left ventricular function through afterload reduction and systemic vasodilation. Mechanistically similar agents, such as the phosphodiesterase III inhibitor milrinone, have also been shown to reduce PCWP in HFpEF patients—particularly during exercise—via enhanced contractility and decreased SBV [36, 37].

Our retrospective analysis evaluated older, real-world HFpEF patients hospitalized with acute HF. Multivariable analysis indicated that the levosimendan group showed significant improvements in septal E/e’ and NYHA class. These findings are consistent with those from the HELP trial [35] and Kass et al. [12], supporting the potential of levosimendan to reduce afterload and enhance functional capacity in HFpEF [38, 39].

## Limitation

As a real-world retrospective study, our research has several inherent limitations:

- First, due to the retrospective design and small sample size, the decision to administer levosimendan was made at the discretion of the attending physician, which may have introduced selection bias. Despite multivariate adjustments, residual confounding from unmeasured factors could persist.
- Second, only patients who underwent echocardiography and BNP reassessment at discharge were included. This inclusion criterion may have led to selection bias, as those who were reassessed might not be fully representative of the entire cohort.
- Third, although echocardiographic reassessment was performed after levosimendan treatment, the timing relative to drug discontinuation varied among patients. The active metabolite of levosimendan has a half-life of approximately 75–80 h, with cardiovascular effects persisting for 7–9 days. This elimination may be further prolonged in patients with renal or hepatic impairment. Unfortunately, we could not measure plasma levels of levosimendan or its active metabolite OR-1896 to accurately account for these pharmacokinetic differences.
- Lastly, measurements of GLS and E/e′ are highly dependent on image quality and are influenced by loading conditions. Although echocardiography was performed consistently, variations in prior treatment (such as diuretic therapy) may have affected preload and consequently the accuracy and interpretation of GLS and E/e′ results.

## Conclusions

For older patients with acute HFpEF combined with more comorbidities, although levosimendan may transiently improve hemodynamics and functional capacity in the acute phase, it fails to demonstrate long-term prognostic benefits. The clinical benefit of this drug may be limited to select patients, and its clinical application may lack cost-effectiveness.

## Data Availability

Data is available upon request to the corresponding author.
